# Effects of Pulsed Electric Field-Assisted Osmotic Dehydration and Edible Coating on the Recovery of Anthocyanins from *In Vitro* Digested Berries

**DOI:** 10.3390/foods8100505

**Published:** 2019-10-17

**Authors:** Gabriel Oliveira, Urszula Tylewicz, Marco Dalla Rosa, Thomas Andlid, Marie Alminger

**Affiliations:** 1 Department of Biology and Biological Engineering, Food and Nutrition Science, Chalmers University of Technology, Kemivägen 10, SE-412 96 Gothenburg, Sweden; thomas.andlid@chalmers.se (T.A.); marie.alminger@chalmers.se (M.A.); 2 Department of Agricultural and Food Sciences, University of Bologna, Piazza Goidanich 60, 74521 Cesena, Italy; urszula.tylewicz@unibo.it (U.T.); marco.dallarosa@unibo.it (M.D.R.)

**Keywords:** anthocyanins, *in vitro* digestion, pulsed electric field, osmotic dehydration, edible coating

## Abstract

Berry fruits, such as strawberries and blueberries, are rich sources of anthocyanins. Several studies have been made on the impact of non-thermal treatments on safety, shelf-life and nutritional characteristics of such products, but the effects of these processes on anthocyanin stability during digestion in the gastrointestinal tract are still not completely clear. The aim of this study was to assess the recovery of anthocyanins after simulated gastrointestinal digestion of (1) strawberry samples, pre-treated with pulsed electric field (PEF) at 100 or 200 V·cm^−1^, prior to osmotic dehydration (OD), and (2) blueberry samples coated with chitosan and procyanidin. After digestion, a significantly higher content of cyanidin-3-*O*-glucoside and malvidin-3-*O*-glucoside was quantified by LC-MS/MS in processed strawberry and blueberry samples, compared with the controls. The highest recovery of cyanidin-3-*O*-glucoside was detected in digested strawberry samples osmotically dehydrated with trehalose. The recovery of malvidin-3-*O*-glucoside was highest in digested blueberries coated with chitosan and stored for 14 days, compared with untreated samples or samples coated with chitosan and procyanidin. Our study shows the potential of mild PEF treatments combined with OD, or the use of edible coating, to obtain shelf-stable products without substantially affecting the composition or the stability of anthocyanins during digestion in the upper gastrointestinal tract.

## 1. Introduction

The consumption of anthocyanin-rich berry fruits has in many scientific studies been suggested to benefit human health [1,2]. Anthocyanins are water soluble pigments responsible for the red, blue and purple colours of fruits, such as strawberries and blueberries. These pigments have been thoroughly investigated due to their high potential to prevent cellular oxidative damage and, consequently, to reduce the risk of inflammatory responses [3].

Anthocyanins are present as glycosides with an anthocyanidin skeleton whose structure undergo chemical changes during human gastrointestinal digestion and are more stable in the acid conditions found in the stomach than under the neutral conditions of the small intestine [4]. The concentration of some anthocyanins can be very low in the intestine [5], and therefore sensitive analytical techniques such as high-performance liquid chromatography (HPLC) coupled with mass spectrophotometry are needed to detect, characterize and quantify these compounds accurately.

Non-thermal processes have gained importance in recent years due to the increasing consumer demand for nutritious, fresh foods, combined with the need for energy-efficient and environmentally friendly processing technologies. Pulsed electric field (PEF) has the potential to minimize detrimental effects on food quality [6]. The application of PEF is based on electrical stimulation pulses that increase the permeabilization of tissues and cells, allowing mass-transfer improvements [7]. As shown in previous studies [8,9], PEF has the potential to enhance the dehydration rate of fruits such as strawberry and kiwi fruit and be applied as a pre-treatment to osmotic dehydration (OD). During the OD process, a counter-current mass transfer occurs, and water is eluted while the solute applied to increase osmotic pressure moves into the cell [10,11]. Pre-treatment using PEF prior to OD treatment can thus be useful to obtain semi-dried fruits.

Another mild processing approach to extend the shelf life of fruits is the application of an edible coating which may create a semi-permeable barrier, improve textural quality, reduce microbial growth and retain the flavour and colour of the fruit [12]. Natural materials such as polysaccharides, plant extracts, protein and lipids have been employed in the formulation of edible coatings [13]. For instance, chitosan combined with procyanidins from grape seeds has been successfully used as an edible coating ingredient to extend the shelf life of blueberries [14,15]. Chitosan, an aminopolysaccharide derived from chitin, possesses antimicrobial properties [16] while procyanidins, polyphenols containing exclusively (epi)catechin, show antioxidant activities [17].

The objectives of the present study were to evaluate the impact of non-thermal processing parameters on the stability of berry anthocyanins during simulated gastrointestinal digestion by assessing the effects of (1) PEF-assisted osmotic dehydration treatments of strawberries to obtain semi-dried products and (2) the use of edible coatings on fresh, whole blueberries.

## 2. Materials and Methods

### 2.1. Chemicals

All chemicals and solvents used were of analytical grade. Methanol (LC-MS grade) was purchased from Fisher Scientific (Loughborough, UK), trifluoroacetic acid and chloroform (HPLC grade) from Sigma-Aldrich Chemical Co. (St. Louis, MO, USA), hydrochloric acid from Acros Organics (Schwerte, Germany), formic acid from Scharlau (Barcelona, Spain), glycerol and citric acid from Sigma-Aldrich (Darmstadt, Germany), Tween 20 from Sigma-Aldrich (Saint Quentin Fallavier, France), sucrose from Eridania Italia SPA (Bologna, Italy) and trehalose from Exacta-Optech Labcenter SPA (San Prospero, Italy). Pepsin from porcine gastric mucosa, pancreatin from porcine pancreas, bile extract from porcine and alfa-amylase from *Bacillus* sp. were purchased from Sigma-Aldrich (Stockholm, Sweden), and chitosan from mushrooms was provided by Agrovin (Alcazar de San Juan, Spain).

Anthocyanin standards (cyanidin-3,5-di-*O*-glucoside chloride, malvidin-3,5-diglucoside, malvidin-3-*O*-glucoside chloride, petunidin-3-*O*-glucose chloride, cyanidin-3-*O*-glucoside chloride, cyanidin-3-*O*-arabinoside chloride, delphinidin-3-*O*-galactoside chloride, and peonidin-3-*O*-glucoside chloride) were all purchased from Extrasynthese (Genay, France).

### 2.2. Raw Materials

**Figure 1:**
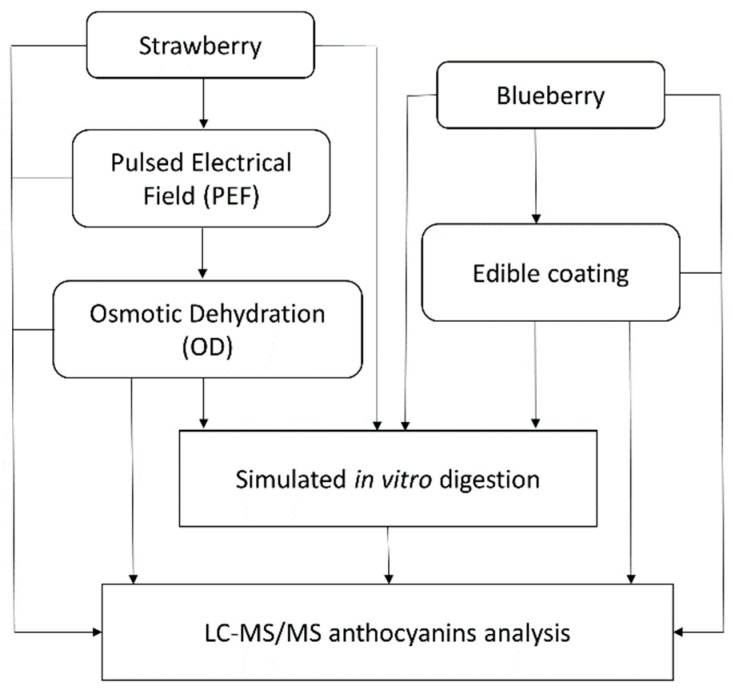
Overview of the materials and methods.

Organic strawberries and blueberries were purchased from the local market. The fruits were kept at 4 ± 1 °C until used (maximum storage time one week). Fresh fruits with similar colour and size and without damage were selected. The strawberries were washed, hand de-stemmed and divided in half along the central axis of the fruit, while blueberries were used as whole berries. Figure 1 shows a schematic overview of the materials and methods used in the study.

### 2.3. Strawberry Samples—Pulsed Electric Field (PEF) Treatment

Prior to osmotic dehydration, strawberry samples (approximately 35 g) were treated with PEF using a pulse generator (S-P7500 60A 8kV, Alintel srl., Bologna, Italy). A rectangular treatment chamber (5 cm × 5 cm × 5 cm) equipped with two stainless steel electrodes (5 cm × 5 cm) with a 5 cm gap between them was used for the experiment. The conductivity of the water was adjusted by sodium chloride to achieve a value of 1.6 mS/cm (measured by EC-Meter basic 30+, Crison, Barcelona, Spain). The following parameters were used for PEF pre-treatment: electric field strength of 100 and 200 V·cm^−1^, rectangular pulses, pulse number of 1000, pulse width of 10 µs, frequency of 100 Hz, and treatment time of 10 s. Pore formation (electroporation) can be reversible or irreversible depending on the treatment intensity. The electric field strength 100 V∙cm^−1^ was selected in order to obtain reversible pores and 200 V∙cm^−1^ for irreversible pores, according to previous reports [8,9]. Field strengths above 200 V∙cm^−1^ where avoided since application of field strengths of 250 and 400 V∙cm^−1^ have been reported to damage cells in apples [18]. Leaching was controlled on the basis of weight loss after the treatment.

### 2.4. Strawberry Samples—Osmotic Dehydration (OD) Treatment

The OD treatment was carried out by immersing the strawberry samples in hypertonic sucrose or trehalose solutions (40%, *w*/*w*) with the addition of 1% (*w*/*w*) of calcium lactate (CaLac) as a structuring agent [8]. The samples were continuously stirred at 25 °C throughout the treatment (120 min). The fruit to solution ratio was 1:4 (*w*/*w*), to avoid concentration changes in the solution during the treatment. 

### 2.5. Blueberry Samples—Edible Coating

Two different coating solutions were prepared, the first one contained 1% (*w*/*w*) chitosan from mushrooms, while the second one was prepared by combining chitosan from mushroom (1%, *w*/*w*) with 0.8% (*w*/*w*) procyanidins (extracted from grape seeds). To each solution, 1.5% (*w*/*w*) glycerol, 0.20% (*w*/*w*) Tween^®^ 20 and 1% citric acid solution were added, as described by Mannozzi et al. [12].

The coating solutions were applied by dipping in two steps (each step 30 s). The first dipping was followed by 60 min of drying at 25 ± 1 °C, and the second by 30 min at the same temperature. Afterwards, coated berry samples were placed in plastic trays (PET), in closed micro-perforated bags (PLA) and stored at 4 °C for 14 days. All blueberry samples were analysed at day 0 and after 14 days of storage.

### 2.6. In Vitro Digestion of Berry Samples

The *in vitro* method simulating digestion was based on Minekus et al. [19], with adaptations. The method consists of three sequential steps: an initial step simulating the oral phase in the mouth, followed by digestion with pepsin/HCl to simulate conditions in the stomach, and a third step with bile salts/pancreatin to simulate conditions in the small intestine. Briefly, in the oral step, 3.0 g of dried berry powder (in triplicate) was mixed with 5 mL of simulated salivary fluid (SSF) and 2.0 g of this mixture was transferred to new tubes. Around 1 g of each sample was saved in a separate tube for moisture analysis. Alfa-amylase solution (5 mL) was added to each tube and vortexed following incubation for 2 min at 37 °C on a rotary shaking plate (250 rpm). In the gastric digestion, 5 mL of pepsin solution was added to the previous tube and the pH was adjusted to 3.0 using HCl (1M). The tube was incubated for 2 h at 37 °C on a rotatory shaking plate (250 rpm). In the intestinal digestion, 5 mL of bile solution and 5mL of pancreatin solution were added to each tube and the pH was adjusted to 7.0 using NaOH (1M). The tube was incubated at 37 °C on a rotatory shaking plate (250 rpm) for 2h. The tubes containing the whole digesta were frozen (−80 °C), freeze-dried and stored at −20 °C until analysis.

### 2.7. Extraction of Anthocyanins

Anthocyanins were extracted according to the methods of Bunea et al. [20] and You et al. [21], with some adaptations. The freeze-dried samples (0.200 g ± 0.015) were mixed by vortexing for 30 s with 5 mL of methanol that contained 0.3% HCl (*v*/*v*) in a glass tube with a screw cap. Nitrogen gas was used to flush the air from the tube. The extraction mixture was placed in the dark at 4 °C for 18 h. After sonication for 15 min at 20 °C, 37 kHz (S15 Elma Sonicator, Elma Schmdbauwer GmbH, Singen, Germany), the samples were centrifuged at 2000× *g* for 10 min and the supernatant fluid was collected. Re-extraction was carried out by adding 5 mL of acidified methanol to the pellet, followed by vortexing, centrifugation and finally pooling the supernatants from each extraction. The supernatants (10 mL) were centrifuged at 4000× *g* for 15 min and stored at −20 °C until analysis.

### 2.8. Determination of Anthocyanins using LC-MS/MS

Anthocyanins were determined by LC-MS/MS analysis using a Shimadzu LC 30AD system combined with a Shimadzu 8030+ MS detector with an electrospray ionization (ESI) source. The separation of anthocyanins was made at 40 °C with a Zorbax Eclipse Plus C18—2.1 mm × 50 mm–1.8 µm column (Agilent Technology, Santa Clara, CA, USA). The mobile phase consisted of aqueous 0.5% formic acid (A) and methanol 0.5% formic acid (B). The flow rate was set at 0.5 mL/min. The samples were eluted using a linear gradient: 0–1 min, 15% B; 1–5 min, 15–50% B; 5–5.1 min, 50–100% B; 5.1–6.0 min, 100% isocratic; 6.0–6.1 min, 100–15% B. The sample injection volume was set at 5 µL. All the MS/MS parameters were optimized by the infusion of each of the standards (1 µg/mL) and the optimal ionization conditions and fragmentation patterns were determined using instrument software (Labsolutions, Shimadzu, Kyoto, Japan). Multiple reaction monitoring (MRM) parameters for each anthocyanin standard are presented in Table 1. In the positive ion mode, the mass spectrometry conditions were as follow: nebulizer gas flow, 2 L/min; drying gas flow, 10 L/min; heating block temperature 400 °C; DL temperature 250 °C; and mass range from 50 to 1200 m/z. Standard calibration curves were constructed for all analytes and the concentration of anthocyanins in the samples was determined using regression analysis (*R*^2^ > 0.99).

### 2.9. Recovery of Anthocyanins

The recovery of anthocyanins in treated strawberry and blueberry samples was estimated by measuring the relative amount of anthocyanins left after *in vitro* digestion. The initial quantity of anthocyanins in the sample before *in vitro* digestion was considered 100%. The recovery of each anthocyanin was calculated as in Equation (1):

(1) $$\mathrm{Recovery}\;\left(\%\right)=\frac{\mathrm{anthocyanin}\;(\mathrm{mg}\cdot {\mathrm{Kg}}^{-1})\;\mathrm{after}\;\mathrm{digestion}}{\mathrm{anthocyanin}\;(\mathrm{mg}\cdot {\mathrm{Kg}}^{-1})\;\mathrm{before}\;\mathrm{digestion}}\times \;100$$

### 2.10. Statistical Analysis

One-way analysis of variance (ANOVA) and Tukey’s Honestly Significant Difference (HSD) test were used to compare the samples. Statistical significance was defined as *p* < 0.05. Statistical analyses were performed with SPSS Statistics ver. 24 software (SPSS Inc., Chicago, IL, USA).

## 3. Results and Discussion

### 3.1. Effect of PEF-Assisted OD on the Recovery of Anthocyanins in Strawberry

Four different anthocyanins were identified and quantified in untreated and treated strawberry samples (Table 2), with cyanidin-3-*O*-glucoside (cy-glc) as the major anthocyanin, which is in agreement with other studies reporting this anthocyanin as one of the most abundant in strawberries [22,23]. The highest content of anthocyanins was found in samples treated with PEF at 200 V·cm^−1^, as a single treatment without OD, while the application of PEF treatment at 100 V·cm^−1^ seemed to have no positive effect on the detectability of anthocyanins, with the exception of peonidin-3-*O*-glucoside (Table 2).

It is well-known that when plant cells are exposed to an external low voltage electric field (<0.5 kV), pores in the membrane may be formed (electroporation), leading to increased permeability and mass transfer in the plant tissue [24]. The level of membrane alteration, including pore formation, depends on the specific characteristics of the membrane as well as the strength of the electric field. For instance, Dellarosa et al. [18] showed that electric field strengths of 250 and 400 V∙cm^−1^ damaged apple cells.

Likely, in the present study, the lower electric field strength (100 V·cm^−1^) yielded reversible electroporation of the cell membrane, which may explain the loss of anthocyanins from cells. On the other hand, at the higher voltage (200 V·cm^−1^), our data suggest that larger and irreversible pores were formed, which may have increased the extractability of the remaining anthocyanins [25].

Table 2 also shows that all the combinations of PEF as a pre-treatment of OD result in lower levels of anthocyanins than in the untreated sample. A hyperosmotic solution causes efflux of water through the plasma-membranes of cells until the isosmotic condition has been reached. However, if the membrane integrity has been disrupted by pores, not only water will leave the cells but all dissolved compounds smaller than the pores, including anthocyanins. This will continue until equilibrium has been reached for all solutes, which explains the consistently lower level of anthocyanins in the berries after PEF has been applied as a pre-treatment of OD (Table 2).

### 3.2. Effect of Edible Coating on the Recovery of Anthocyanins in Blueberry Samples

Table 3 shows the amount of anthocyanins (mg.Kg^−1^, DW) quantified in uncoated blueberries (F) and in blueberries coated with chitosan (C) or chitosan and procyanidin from grape seeds (Cp). The most abundant anthocyanin found in blueberry was malvidin-3-*O*-glucoside (ma-glc), followed by delphinidin-3-*O*-galactoside and petunidin-3-*O*-glucoside. For all samples (coated and uncoated), storage for 14 days (T14) increased the concentration of anthocyanins in the fruit (Table 3). The observed increase is likely due to ripening-related accumulation of anthocyanins [26,27].

### 3.3. Recovery of Anthocyanins from Processed Strawberry after In Vitro Digestion

Anthocyanins are usually unstable during digestion, especially in the small intestine, where they are mostly transformed into different monomeric phenolic acids and aldehydes [4]. Moreover, reaching the large intestine, microbiota can play an important role in the metabolism of anthocyanins showing prebiotic activities [28,29]. Therefore, increasing stability of anthocyanins during digestion may result in higher availability of these compounds to the gut microbiota.

**Figure 2:**
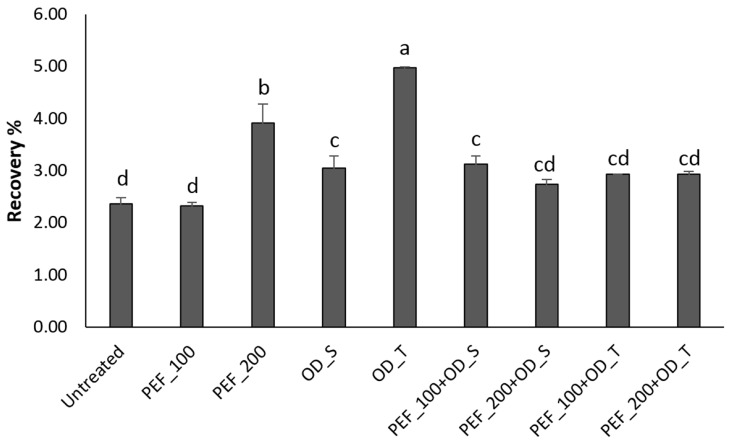
Relative recovery of cyanidin-3-*O*-glucoside after *in vitro* digestion of untreated and treated strawberry samples. Different letters indicate significant differences (*p* < 0.05) between treatments, based on Tukey’s tests (*n* = 3). PEF refers to pulsed electric field, at 100 or 200 V·cm^−1^, and OD refers to osmotic dehydration with sucrose (S) or trehalose (T).

**Figure 3:**
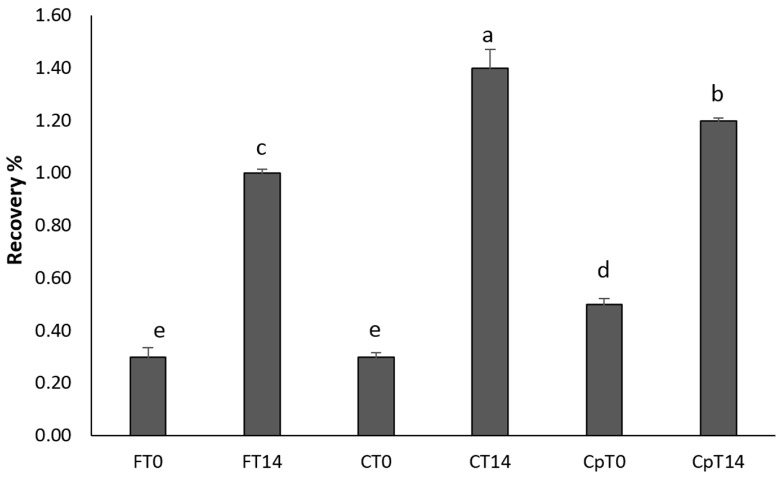
The combined recoveries of malvidin-3-*O*-glucoside from untreated and treated blueberry samples. Different letters indicate significant differences (*p* < 0.05) between treatments, based on Tukey’s tests (*n* = 3). F refers to uncoated samples, C to samples coated with chitosan and Cp to samples coated with chitosan and procyanidins. (T0) without storage and (T14) after storage for 14 days.

In our study, after digestion of the treated samples, a significantly higher content of anthocyanins could be quantified in strawberry (cyanidin-3-*O*-glucoside) and in blueberry (malvidin-3-*O*-glucoside) compared with the non-treated samples, with recoveries ranging from 2.5% to 5% and 0.3% to 1.5%, respectively (Figure 2 and Figure 3). A higher recovery was found for cyanidin-3-*O-*glucoside likely due to higher stability of this compound under intestinal conditions compared with malvidin-3-*O*-glucoside [30]. Tagliazucchi et al. [31] reported a recovery of anthocyanins of 7% after *in vitro* gastrointestinal digestion of plums. On the other hand, Kamiloghu et al. [32] found a recovery of anthocyanins ranging from 0.1% to 58.1% after *in vitro* gastrointestinal digestion of black carrot (*Daucus carota*) jams and marmalades. It is worthwhile to mention that, in an aerobic *in vitro* digestion system performed in open-air tubes, as applied in the present study, the degradation of polyphenols may be overestimated due to the considerable sensitivity of polyphenols to oxygen [33]. Therefore, the low recoveries of malvidin and cyanidin glucosides found in our study are in line with previous *in vitro* studies and may be due to the oxidative degradation of anthocyanins [31,32,33].

The recovery of cy-glc in strawberry samples treated with PEF at 200 V·cm^−1^ was 37% higher than in untreated samples or samples treated with PEF at 100 V·cm^−1^ (Figure 2). This is likely due to an enhanced extractability in samples treated with PEF 200, as a consequence of larger and more stable pores created in the cell membranes during the treatment at a higher electric field strength. Even though the pores formed after PEF at 200 V∙cm^−1^ may lead to losses and degradation of some anthocyanins during digestion, the processing may also promote the release of intracellular compounds and, hence, enable a higher recovery of anthocyanins.

Although osmotic dehydration with trehalose (OD_T) markedly reduced the concentration of cy-glc (Table 2) compared with the control (untreated), after digestion this anthocyanin showed the highest recovery (Figure 2). It is known that trehalose preserves lipid bilayers during dehydration and protects biomolecules [34,35,36]. According to Tang et al. [37] one possible explanation for this protective effect is that trehalose, by replacing water to form hydrogen bonds between its own OH groups and lipid headgroups, preserves lipid bilayers during dehydration. Anthocyanins are localized inside vacuoles in the plant cells where these pigments are protected [38]. Hence, trehalose may in our experiments have stabilized the cell and vacuole membranes, and consequently limited the degradation of cy-glc during the digestion. On the other hand, using sucrose to raise the osmolarity in the surrounding solution resulted in a lower recovery of cy-glc compared with the use of trehalose (Figure 2). Trehalose has a larger flexibility between its two monomers compared with the monomers of sucrose, which has been suggested to improve interactions with other molecules [39]. Further investigations are needed to understand the effects of different sugar-based matrices on the protection of anthocyanins during *in vitro* digestion.

### 3.4. Recovery of Anthocyanins from Coated Blueberry after In Vitro Digestion

Edible coating is a promising technique for preservation of fruits and berries. This is especially interesting when natural compounds, such as chitosan, are used as a coating matrix. Chitosan is an aminopolysaccharide formed by the deacetylation of chitin, commonly found as a component of the exoskeleton of insects and crustaceans, as well as in cell walls of most fungi. Chitosan cannot be enzymatically cleaved in the human body but has been found safe and applicable as a food ingredient [40,41].

There was a notable difference between the recovery of ma-glc from *in vitro* digested blueberry samples coated with only chitosan and stored (FT14, Figure 3), and the uncoated and stored blueberries (CT14, Figure 3). The use of chitosan as a single ingredient may contribute to a more stable coating than when combined with procyanidins (Cp14, Figure 3), and hence enhance the protection of the anthocyanins during digestion. A protective effect of anthocyanins by chitosan in simulated gastrointestinal fluids has been reported by He et al. [42], who showed that, compared to preparations containing free anthocyanins, loading of anthocyanins on chitosan nanoparticles resulted in slower degradation and improved stability of anthocyanins in a model beverage system. Chitosan is a cationic molecule [41] and may by ionic interactions with anthocyanins increase the stability of some structures and prevent degradation.

Blueberry samples coated with chitosan combined with procyanidins (Cp0, Figure 3) had a higher recovery of ma-glc directly after coating than after storage for 14 days, suggesting that this combination of coating ingredients may be unstable during storage.

It is important to take into account that even though procyanidins and anthocyanins belong to the same family of polyphenolic compounds (flavonoids), the former exists primarily as glucosides while procyanidins are usually present as aglycons [43]. Procyanidins are a subgroup of condensed tannins containing exclusively (epi)catechin [17,44]. According to previous HPLC MS-MS data analysis, MS fragments of procyanidins and anthocyanins, as well as their retention times, are different, thus limiting the possibility of interference during analyses of these compounds [23,45].

## 4. Conclusions

The application of pulsed electric field (PEF)-assisted osmotic dehydration (OD) to strawberries and the use of edible coatings of blueberries maintained or enhanced the stability of anthocyanins during gastrointestinal *in vitro* digestion. The recovery of cy-glc, the main anthocyanin detected in strawberry samples after digestion, was higher after PEF treatment at 200 V·cm^−1^ compared with PEF 100 V·cm^−1^, untreated samples and PEF-assisted OD.

Strawberries osmotically dehydrated with trehalose had the highest recovery of cy-glc while the recovery of ma-glc was the highest in blueberries coated with chitosan and stored for 14 days.

Our results suggest that trehalose and chitosan work as protective compounds reducing the degradation of cy-glc and ma-glc, respectively, during digestion in the upper gastrointestinal tract. However, further studies are needed to elucidate the mechanisms and potential interactions of anthocyanins with disaccharides and chitosan. In the current study strawberries and blueberries were also used as model fruits for evaluation of two different technologies to preserve their quality and extend their shelf-life. More research is needed to compare the results with different food matrices, by application of the processing methods used in this study on different fruits and vegetables.

PEF at 200 V∙cm^−1^ and chitosan-based edible coating were found promising as energy efficient and mild processing techniques to obtain berry products with a maintained content of anthocyanins and higher stability during digestion.

## Figures and Tables

**Table 1:** Optimized multiple reaction monitoring (MRM) conditions for LC-MS/MS analyses of anthocyanins.

Anthocyanin	Retention Time (min.)	MRM Transitions (*m/z*) [M + H]^+^	Collision Energy (V)
cyanidin-3,5-di-*O*-glucoside	1.3	611.00 → 287.05	37
malvidin-3,5-diglucoside	2.4	655.10 → 331.00	35
malvidin-3-*O*-glucoside	4.7	493.00 → 331.00	33
petunidin-3-*O*-glucoside	3.5	478.90 → 317.05	23
cyanidin-3-*O*-arabinoside	3.0	418.90 → 287.00	20
peonidin-3-*O*-glucoside	3.3	462.90 → 301.00	22
delphinidin-3-*O*-glucoside	2.1	465.20 → 303.00	22
cyanidin-3-*O*-glucoside	2.8	448.90 → 287.12	23

**Table 2:** Anthocyanins quantified in untreated and treated strawberry samples before *in vitro* digestion.

Sample	Anthocyanins mg·Kg^−1^ DW			
	Cyanidin-3-*O*-Glucoside	Petunidin-3-*O*-Glucoside	Cyanidin-3-*O*-Arabinoside	Peonidin-3-*O*-Glucoside
Untreated	91.75 ± 5.48 ^b^	0.61 ± 0.01 ^a^	0.037 ± 0.001 ^bc^	0.36 ± 0.02 ^bc^
PEF_100	90.50 ± 5.51 ^b^	0.37 ± 0.02 ^d^	0.026 ± 0.001 ^cde^	0.41 ± 0.03 ^b^
PEF_200	107.15 ± 4.16 ^a^	0.56 ± 0.03 ^ab^	0.054 ± 0.004 ^a^	0.55 ± 0.01 ^a^
OD_S	81.99 ± 4.33b ^c^	0.33 ± 0.01 ^d^	0.037 ± 0.003 ^bc^	0.34 ± 0.01 ^bcd^
OD_T	66.56 ± 5.20 ^de^	0.47 ± 0.01 ^c^	0.057 ± 0.005 ^a^	0.37 ± 0.01 ^b^
PEF_100 + OD_S	63.76 ± 0.67 ^e^	0.35 ± 0.04 ^d^	0.020 ± 0.003 ^de^	0.37 ± 0.03 ^b^
PEF_200 + OD_S	73.51 ± 1.89 ^cd^	0.38 ± 0.02 ^d^	0.031 ± 0.002 ^bcd^	0.27 ± 0.01 ^d^
PEF_100 + OD_T	79.14 ± 1.80 ^c^	0.38 ± 0.01 ^d^	0.039 ± 0.001 ^b^	0.39 ± 0.02 ^b^
PEF_200 + OD_T	75.17 ± 4.54 ^cd^	0.35 ± 0.02 ^d^	0.016 ± 0.001 ^e^	0.30 ± 0.01 ^cd^

PEF_100 and _200 refer to a pulsed electric field at 100 or 200 V·cm^−1^, respectively, and OD refers to osmotic dehydration with sucrose (S) or trehalose (T). Values followed by the same letter in each column were not significantly different (*p* < 0.05) based on Tukey’s tests.

**Table 3:** Anthocyanin quantified in uncoated and coated blueberry samples before *in vitro* digestion.

Sample	Anthocyanins mg·Kg^−1^ DW		
	Cyanidin-3-*O*-Glucoside	Malvidin-3-*O*-Glucoside	Petunidin-3-*O*-Glucoside
FT0	285.06 ± 14.41 ^c^	3115.57 ± 6.72 ^b^	1608.55 ± 103.11 ^d^
FT14	345.98 ± 14.65 ^ab^	3397.17 ± 97.75 ^a^	1897.46 ± 24.70 ^bc^
CT0	210.87 ± 10.78 ^d^	2519.57 ± 99.88 ^c^	1223.83 ± 46.11 ^e^
CT14	355.21 ± 17.17 ^a^	3529.05 ± 18.77 ^a^	2121.66 ± 58.58 ^ab^
CpT0	297.71 ± 9.56 ^c^	2528.33 ± 112.29 ^c^	1685.97 ± 77.91 ^cd^
CpT14	310.04 ± 6.47b ^c^	3547.34 ± 84.20 ^a^	2237.28 ± 107.31 ^a^
	Cyanidin-3-*O*-Arabinoside	Peonidin-3-*O*-Glucoside	Delphinidin-3-*O*-Galactoside
FT0	163.25 ± 4.62 ^b^	144.27 ± 1.20 ^c^	1715.19 ± 21.22 ^c^
FT14	198.91 ± 1.70 ^a^	171.49 ± 3.97 ^b^	2134.96 ± 98.39 ^ab^
CT0	167.62 ± 9.92 ^b^	114.45 ± 3.36 ^d^	1389.58 ± 1.96 ^d^
CT14	194.50 ± 0.31 ^a^	163.77 ± 1.12 ^b^	2313.76 ± 118.86 ^a^
CpT0	159.81 ± 11.08 ^b^	122.31 ± 3.68 ^d^	2020.33 ± 10.24 ^b^
CpT14	197.04 ± 3.99 ^a^	193.75 ± 9.61 ^a^	2117.93 ± 54.36 ^ab^

Values followed by the same letter in each column were not significantly different (*p* < 0.05) based on Tukey’s tests.
